# Ruxolitinib versus dexamethasone in hospitalized adults with COVID-19: multicenter matched cohort study

**DOI:** 10.1186/s12879-021-06982-z

**Published:** 2021-12-22

**Authors:** O. V. Stanevich, D. S. Fomina, I. G. Bakulin, S. I. Galeev, E. A. Bakin, V. A. Belash, A. N. Kulikov, A. A. Lebedeva, D. A. Lioznov, Yu. S. Polushin, I. V. Shlyk, E. A. Vorobyev, S. V. Vorobyeva, T. V. Surovceva, N. V. Bakulina, M. A. Lysenko, I. S. Moiseev

**Affiliations:** 1 Pavlov University, Saint-Petersburg, Russian Federation; 2 State City Hospital №52, Moscow, Russian Federation; 3 North-Western State Medical University Named After I.I. Mechnikov, Saint-Petersburg, Russian Federation; 4 State City Hospital №20, Saint-Petersburg, Russian Federation; 5 First Sechenov Moscow State Medical University of the Ministry of Healthcare of the Russian Federation, Moscow, Russia; 6 Pirogov Russian National Research Medical University (RNRMU) of the Ministry of Healthcare of the Russian Federation, Moscow, Russia

**Keywords:** COVID-19, SARS-CoV-2, Ruxolitinib, Dexamethasone, Anti-cytokine therapy

## Abstract

**Background:**

Several anti-cytokine therapies were tested in the randomized trials in hospitalized patients with severe acute respiratory syndrome coronavirus 2 infection (COVID-19). Previously, dexamethasone demonstrated a reduction of case-fatality rate in hospitalized patients with respiratory failure. In this matched control study we compared dexamethasone to a Janus kinase inhibitor, ruxolitinib.

**Methods:**

The matched cohort study included 146 hospitalized patients with COVID-19 and oxygen support requirement. The control group was selected 1:1 from 1355 dexamethasone-treated patients and was matched by main clinical and laboratory parameters predicting survival. Recruitment period was April 7, 2020 through September 9, 2020.

**Results:**

Ruxolitinib treatment in the general cohort of patients was associated with case-fatality rate similar to dexamethasone treatment: 9.6% (95% CI [4.6–14.6%]) vs 13.0% (95% CI [7.5–18.5%]) respectively (*p* = 0.35, OR = 0.71, 95% CI [0.31–1.57]). Median time to discharge without oxygen support requirement was also not different between these groups: 13 vs. 11 days (*p* = 0.13). Subgroup analysis without adjustment for multiple comparisons demonstrated a reduced case-fatality rate in ruxolitnib-treated patients with a high fever (≥ 38.5 °C) (OR 0.33, 95% CI [0.11–1.00]). Except higher incidence of grade 1 thrombocytopenia (37% vs 23%, *p* = 0.042), ruxolitinib therapy was associated with a better safety profile due to a reduced rate of severe cardiovascular adverse events (6.8% vs 15%, *p* = 0.025). For 32 patients from ruxolitinib group (21.9%) with ongoing progression of respiratory failure after 72 h of treatment, additional anti-cytokine therapy was prescribed (8–16 mg dexamethasone).

**Conclusions:**

Ruxolitinib may be an alternative initial anti-cytokine therapy with comparable effectiveness in patients with potential risks of steroid administration. Patients with a high fever (≥ 38.5 °C) at admission may potentially benefit from ruxolitinib administration.

*Trial registration* The Ruxolitinib Managed Access Program (MAP) for Patients Diagnosed With Severe/Very Severe COVID-19 Illness NCT04337359, CINC424A2001M, registered April, 7, 2020. First participant was recruited after registration date

**Supplementary Information:**

The online version contains supplementary material available at 10.1186/s12879-021-06982-z.

## Background

It is now established that excessive production of cytokines is an important part of pathogenesis during severe acute respiratory syndrome coronavirus 2 (SARS-CoV-2) infection (COVID-19) [1, 2]. Usually patients present with an elevation of multiple cytokines [3, 4]. Clinical consequence of increased cytokine production is the development of hyperinflammatory syndrome (HIS), also called cytokine-release syndrome, or macrophage-activation syndrome, or secondary haemophagocytic lymphohistiocytosis. Symptoms of HIS usually include a high fever (≥ 38.5 °C), disseminated intravascular coagulation (DIC), acute respiratory distress syndrome (ARDS), encephalopathy, abnormal liver function tests, acute kidney injury, lymphopenia, low platelet count [2, 5].

Based on these observations of HIS, various types of anti-cytokine therapies were evaluated in COVID-19 patients, including anti-interleukin-6 (IL-6) [6], anti-interleukin-1 (IL-1) [7] and anti-granulocyte-monocyte colony-stimulating factor (GM-CSF) [8]. Despite a faster resolution of symptoms, which was reported in these studies so far, only dexamethasone [9] and baricitinib [10], a Janus kinase (JAK) 1 and 2 inhibitor, demonstrated improved survival in patients hospitalized with COVID-19 compared to the standard of care.

Another JAK 1/2 inhibitor, ruxolitinib, also demonstrated effective anti-cytokine properties in myelofibrosis [11], graft-versus host disease (GVHD) [12] and secondary haemophagocytic lymphohistiocytosis [13]. Also, ruxolitinib was reported to improve COVID-19 clinical course in several small patient series [14, 15]. Recently the press release indicated that randomized RUXCOVID study did not meet its primary endpoint of reduced cumulative incidence of death, mechanical ventilation, or ICU care compared to the standard of care [16]. Since the ‘standard’ of care for COVID-19 is constantly changing and, unlike the early studies, now a majority of severe patients do receive some form of anti-cytokine therapy, we compared ruxolitinib with the most common immunosuppressive treatment, dexamethasone, in the multicenter matched cohort study.

## Objective

Estimation of ruxolitinib effectiveness and safety in comparison with conventional glucocorticosteroid therapy in COVID-19 patients.

## Methods

### Patients

**Fig. 1:**
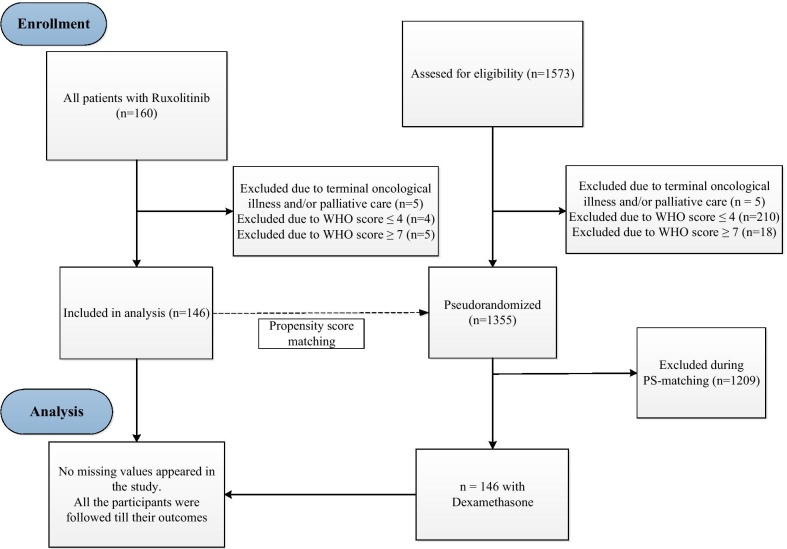
Participant flow diagram with enrollment and analysis procedures in the study

The study was conducted in four large specialized hospitals for COVID-19 infection in Saint-Petersburg and Moscow. Recruitment period was April 7, 2020 through September 9, 2020. The active treatment arm included 146 patients (Fig. 1) from the Ruxolitinib Managed Access Program (MAP) for Patients Diagnosed With Severe/Very Severe COVID-19 Illness (www.clinicaltrials.gov NCT04337359, CINC424A2001M), recruited after the date of the trial registration. The inclusion criteria in this analysis were PCR-confirmed case of COVID-19 infection, respiratory support and 5–6 score on the Ordinal scale [17, 18]. Patients with scores 4 and less were excluded because these patients generally receive outpatient care in the Russian Federation and the sample of hospitalized patients is not representative of this population. Patients with score 7 and higher were excluded because there is limited data on pharmacokinetics of ruxolitinib after administration of crushed pills via gastric tube. The only exclusion criterion in the analysis was a terminal oncological illness on a palliative care. All consecutive patients meeting the inclusion and exclusion criteria were included in the analysis.

COVID-19 infection was confirmed by both the nasopharyngeal and oropharyngeal swab with RealBest RNA SARS-CoV-2 test kit for real time PCR (Vector-Best, Novosibirsk, Russian Federation). Patients were followed up until death or discharge. Local ethical committees approved the participation of patients in the Managed Access Program and all patients signed informed consent for the treatment. Ruxolitinib was administered in doses 5–10 mg bid. Median dose was 0.125 was mg/kg/day. Dose distribution per kilogram of patient weight is presented in Additional file 1: Fig. S1. Ruxolitinib was administered until oxygen support withdrawal. Computer tomography (CT) severity grade was assessed using the recommendations by the Russian Ministry of Health. In brief, the grading system is based on the percentage of affected lung tissue: grade 1 (< 25%), grade 2 (25–50%), grade 3 (51–75%), grade 4 (> 75%) [17].

The control group was selected from 1355 simultaneously hospitalized patients (Fig. 1) receiving 16–24 mg of dexamethasone daily due to respiratory failure (WHO score > 4). The duration of dexamethasone therapy was 5–10 days based on the symptoms severity.

Data was downloaded from the local health information system and manually checked for potential outliers. There were no missing values in baseline data. For the restoration of unknown values between two sequential tests, the “last observation carried forward” procedure (LOCF) was applied.

### Outcome measures, group matching and statistical analysis

The following major clinical and laboratory disease features were used for selection of patients for the control group: age, gender, body mass index (BMI), SpO_2_ without oxygen support, C-reactive protein (CRP), pre-existing diabetes, absolute lymphocyte count and the day of illness. Additional parameters assessed during matching were body temperature, serum ferritin, serum creatinine, serum glucose level, white blood cell (WBC) count, potassium, sodium, hemoglobin, neutrophil count, monocyte count, platelet count, pre-existing HIV, cardiovascular, respiratory, liver disorders, tuberculosis in the patient’s history and oncological disease requiring active treatment. CT grade was not included in the matching parameters because it was not yet validated for patient case-fatality. Only pre-treatment laboratory measures were used for matching. The propensity score matching was based on all of these parameters and selection of the control group with 1:1 ratio. The balance of covariates before and after matching is given in Additional file 1: Fig. S2. Contingency table for case-fatality rate before and after matching is available in Additional file 1: Table S1.

The World Health Organization (WHO) recommendations for selection of outcomes were used to define the outcome of the study [19]. Cumulative incidences of death and discharge as competing risks were selected as the primary outcome of the study based on the following considerations: (1) the universal healthcare system in the Russian Federation excludes discharges for economic reasons, which is the main concern about discharge endpoint; (2) we were not limited in the follow up time of intubated or discharged patients, so either one or another outcome did occur within the scope of the study; (3) time to PCR-negativity was not an appropriate endpoint in this study because some asymptomatic patients were discharged before PCR-negativity. The differences in clinical measures, laboratory parameters and toxicity between study groups were assessed Chi-square and Mann–Whitney tests according to the data type. Cumulative incidence analysis and Gray test were used to compare case-fatality rates and discharge rates between dexamethasone and ruxolitinib arms.

Also a subgroup analysis based on binary thresholding of various variables was performed. In it odds ratios derived from 2 × 2 contingency tables with death as an outcome were estimated. CRP threshold for subgroup analysis was selected from the set of dexamethasone patients not included in the main analysis. In this group of patients receiver operating curve (ROC) analysis was performed targeting highest average sensitivity and specificity for in-hospital case-fatality rate. The resulting threshold level was approximately 100 mg/L (Additional file 1: Fig. S4). All analyses were performed using *R* software, version 4.0.4. The packages used for the analysis are listed in Additional file 1: Table S4.

For sensitivity analysis we conducted a clustering of ruxolitinib group based on the selected matching variables. The survival in the three selected clusters was 85% vs 98% vs 87%, *p* = 0.0087 (Additional file 1: Fig. S4, Table S2). Thus, the algorithm effectively selected the cluster 2 of patients with a low risk of death. After that for each cluster a matching was performed for a control group selection and a comparison of case fatality rates (Additional file 1: Fig. S5, Table S3).

## Results

Analyzed groups of patients with ruxolitinib (N = 146) and dexamethasone (N = 146) treatments were well matched in their clinical and laboratory parameters (Table 1). All patients included in the study received intermediate doses of anticoagulant medications (low-molecular-weight heparin). In the ruxolitinib group, 94% of patients were treated with antibiotic therapy, in the control group—88%. For 32 patients from ruxolitinib group (21.9%) with ongoing progression of respiratory failure after 72 h of treatment, additional anti-cytokine therapy was prescribed (8–16 mg dexamethasone).

**Table 1:** Characteristics of patients treated with ruxolitinib and matched control group treated with dexamethasone

Names	Reference arm (*n* = 146)	Treatment arm (*n* = 146)	*p*-value
Age, years, mean ± SD	58 ± 12.9	58.1 ± 13.3	0.93
Gender	F: 64 (43.8%) M: 82 (56.2%)	F: 66 (45.2%) M: 80 (54.8%)	0.81
Body mass index, mean ± SD	30.7 ± 13.4	30.4 ± 5.6	0.81
Day of illnes	7.4 ± 4.3	7.5 ± 4.3	0.87
Admission creatinine, mmol/L	0.1 ± 0	0.1 ± 0	0.67
Admission CRP, mg/L	99.5 ± 84.7	97.4 ± 71.9	0.82
Maximal CRP, mg/L	105.2 ± 80.6	101.1 ± 86.4	0.68
Admission ferritin, ng/mL	646 ± 538.3	617 ± 403.8	0.6
Maximal glucose, mmol/L	7.7 ± 2.7	7.6 ± 2.9	0.71
Maximal temperature, °C	38.1 ± 0.8	38.1 ± 0.9	0.77
Mean lymphocytes, 109/L	1.2 ± 0.5	1.3 ± 0.8	0.074
Mean monocytes, 109/L	0.4 ± 0.2	0.5 ± 0.3	0.41
Mean neutrophils, 109/L	5.3 ± 3.8	5.1 ± 3.1	0.72
Mean potassium, mmol/L	4 ± 0.4	4 ± 0.5	0.95
Mean sodium, mmol/L	138.7 ± 3.4	138.6 ± 3.8	0.83
Mean WBC, 109/L	6.9 ± 3.8	6.8 ± 3.2	0.79
Admission hemoglobin, g/L	133.5 ± 18.7	134.4 ± 19	0.69
Minimal platelets, 109/L	202.2 ± 68.9	201.1 ± 64.2	0.89
Admission SpO_2_, %	93.7 ± 2.3	93.6 ± 3.2	0.74
Minimal SpO_2_, %	93.8 ± 3.3	93.5 ± 4.6	0.43
Cardiovascular disease	Yes: 75 (51.4%) No: 71 (48.6%)	Yes: 73 (50%) No: 73 (50%)	0.82
Diabetes	Yes: 33 (22.6%) No: 113 (77.4%)	Yes: 29 (19.9%) No: 117 (80.1%)	0.57
HIV	Yes: 2 (1.4%) No: 144 (98.6%)	Yes: 1 (0.7%) No: 145 (99.3%)	0.56
Chronic liver disease	Yes: 5 (3.4%) No: 141 (96.6%)	Yes: 5 (3.4%) No: 141 (96.6%)	1
Oncological disease not in remission	Yes: 7 (4.8%) No: 139 (95.2%)	Yes: 9 (6.2%) No: 137 (93.8%)	0.61
Chronic respiratory disease	Yes: 11 (7.5%) No: 135 (92.5%)	Yes: 17 (11.6%) No: 129 (88.4%)	0.23
Tuberculosis in the patient’s history	Yes: 4 (2.7%) No: 142 (97.3%)	Yes: 3 (2.1%) No: 143 (97.9%)	0.7
Severity of lung injury based on computed tomography	CT-1: 16 CT-2: 83 CT-3: 47	CT-1: 20 CT-2: 97 CT-3: 29	0.058
Additional antibiotic therapy	Yes: 128 (88%) 69 (54%)—azithromycin 10 (8%)—beta-lactam antibiotics 10 (8%)—fluorchinolone 38 (30%)—antibacterial combination^a^ No: 18 (12%)	Yes: 137 (94%) 40 (29%)—azithromycin 15 (11%)—beta-lactam antibiotics 9 (7%)—metronidazole 73 (53%)—antibacterial combination^b^ No: 9 (6%)	0.105

All laboratory values include levels before either ruxolitinib or dexamethasone administration. Laboratory values are presented as mean ± standard deviation

*CRP* C-reactive protein, *HIV* human immunodeficiency virus infection

^a^Antibacterial drugs combination (azithromycin + beta-lactams, carbapenems + fluorchinolone, beta-lactams + metronidazole, etc.)

^b^Antibacterial drugs combination (azithromycin + beta-lactams, carbapenems + fluorchinolone, vancomicyn + metronidazole, etc.)

**Fig. 2:**
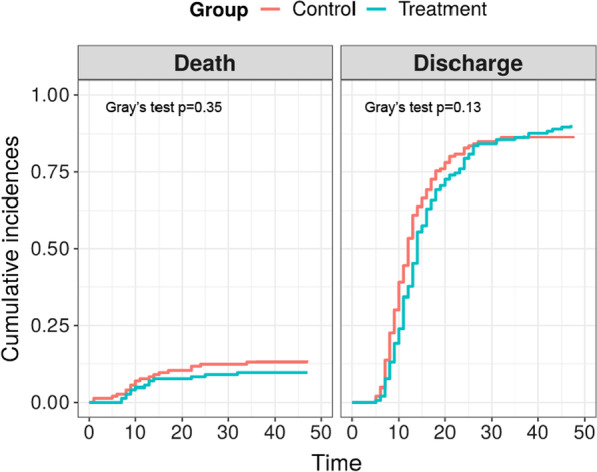
Cumulative incidence of death and discharge in the dexamethasone (control) and ruxolitinib groups (treatment)

**Fig. 3:**
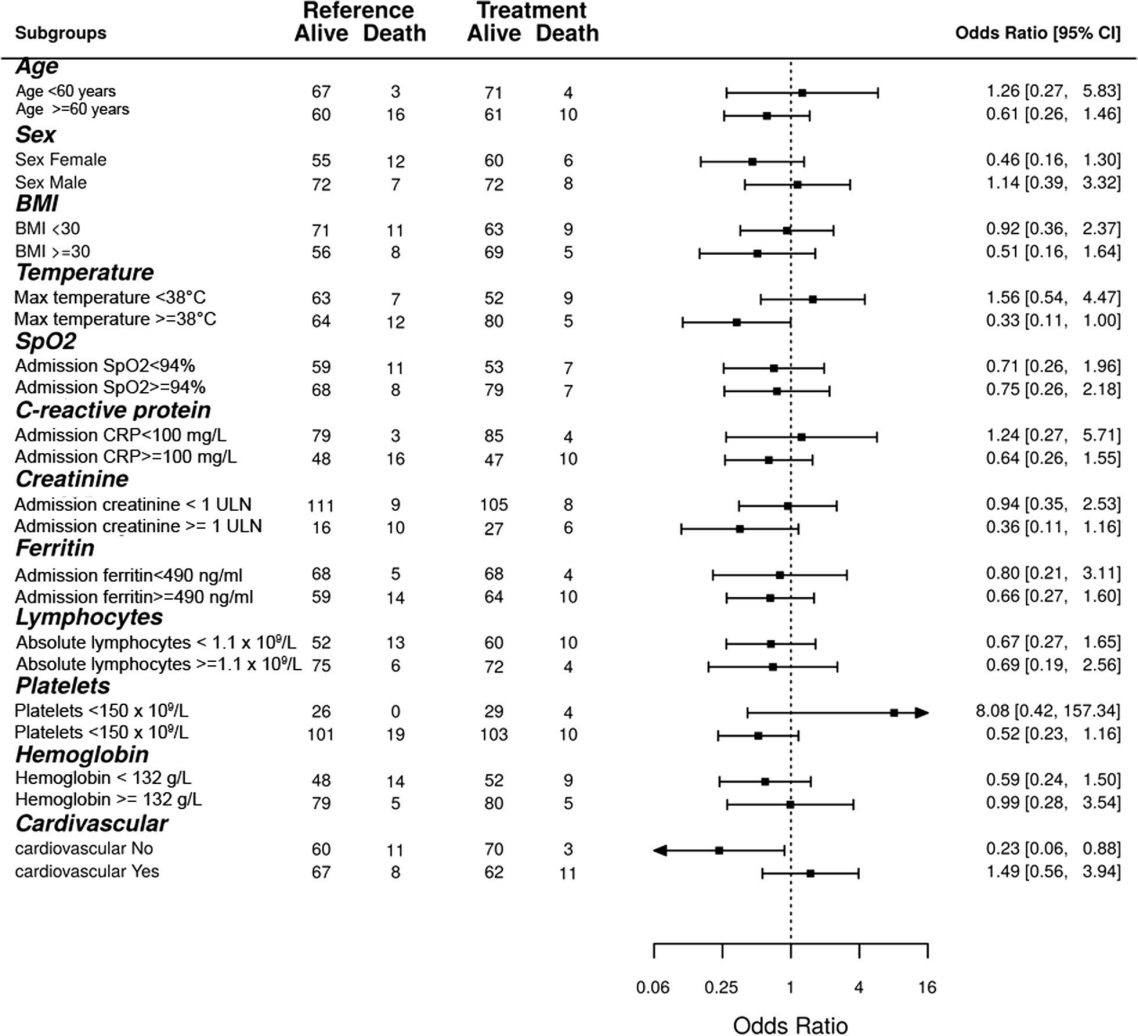
Forrest plot with subgroup analysis of case-fatality rates according to the clinical and laboratory characteristics. Bars to the left of the reference line indicate superiority of ruxolitinib, to the right—superiority of dexamethasone. ULN = upper limit of normal. Max = maximal value documented in-hospital before anti-cytokine therapy. The cut off levels of absolute lymphocytes and hemoglobin represent local normal reference ranges

Cumulative incidence of death from COVID-19 was 9.6% in the ruxolitinib group (N = 14) and 13.0% in the dexamethasone group (N = 19), *p* = 0.35, OR = 0.71, 95% CI [0.31–1.57] (see Fig. 2). Absolute risk difference—3.4% (95% CI [− 3.8, 10.7%]). Median time to discharge was 13 vs 11 days for these groups, respectively (*p* = 0.13). The subgroup analysis of case-fatality rate without adjustment for multiple comparisons demonstrated that dexamethasone was not superior to ruxolitinib in any of the subgroups tested. However, ruxolitinib administration was associated with a borderline case-fatality reduction in patients with a high persistent fever (OR 0.33, 95% CI 0.11–1.00). Also, surprisingly, improved survival was observed not in patients with cardiovascular disease who were expected to tolerate steroids worse than ruxolitinib, but in patients without cardiovascular disease (OR 0.23, 95% CI 0.06–0.88, Fig. 3). None of the other complete blood count parameters (Additional file 1: Fig. S6), biochemistry parameters (Additional file 1: Fig. S7), demographic parameters (Additional file 1: Fig. S8) or CT stage (Additional file 1: Table S5) were predisposing to better survival with either of the anti-cytokine therapies.

The analysis of complications demonstrated almost equal distribution of hematological adverse events between groups (Additional file 1: Table S6). Only grade 1 thrombocytopenia was observed more often after ruxolitinib (37% vs 23%, *p* = 0.042). The incidence of grade 2–4 thrombocytopenia was not different between the groups (4.1% vs 3.4%). However, fewer severe cardiovascular adverse events were observed in the ruxolitinib group (6.8% vs 15%, *p* = 0.025), including reduction in the incidence of pulmonary embolism (2.0% vs 7.5%, *p* = 0.028) and trend to decreased incidence of acute myocardial infarction (4.8% vs 10.3%, *p* = 0.076). Incidence of deep vein thrombosis was not different between two arms (1.4% vs 1.4%, *p* = 1.0). Overall incidence of secondary severe infectious adverse events was not different between groups (7.5% vs 9.6%, *p* = 0.53). The incidence of nosocomial bacterial pneumonias (3.4% vs 4.1, *p* = 0.76) and sepsis (4.1% vs 6.1%, *p* = 0.43) was also not different in the ruxolitinib and dexamethasone groups, respectively. The list of rare adverse events is presented in Additional file 1: Table S7. All rare adverse events occurred no earlier than day 5 of the ruxolitinib treatment. In three cases (toxic hepatitis grade 3, gastrointestinal bleeding and spontaneous intra-abdominal hemorrhage) the therapy was changed to the conventional dexamethasone treatment (16–24 mg).

## Discussion

Although WHO does not recommend routine use of immunosuppressive therapies [20], dexamethasone [9] and JAK inhibitor, baricitinib [10], were the few agents that demonstrated improvement of survival in large populations of patients with severe COVID-19 infection. In our study in the general group there were no significant differences between the evaluated agents in prevention of death from COVID-19, however dexamethasone administration was associated with faster hospital discharge. Despite absence of survival advantage in the general population, during the matching process we observed that COVID-19 is a very heterogeneous disease. This might be the reason for the failure of several randomized trials [6, 21]. Thus in attempts to find a universal cure for all COVID-19 patients it is important to formulate which patient population is the candidate for immunosuppressive therapy and what kind of anti-cytokine therapy should be applied in different clinical situations.

It is clear that at least in part this heterogeneity in COVID-19 patients comes from HIS clinical presentation. Before COVID-19 pandemic, it was evident that each etiology of HIS, or CRS, is associated with unique clinical features. CRS after chimeric antigen receptor T cells is characterized by neurotoxicity and hypotension [22]. In rheumatic diseases most common clinical features are hepatomegaly, splenomegaly, polyserositis and cytopenias [23]. In sepsis and hematological malignancies it is multiorgan failure with overproduction of serum ferritin [24, 25]. HIS in severe COVID-19 is another distinct entity with mild to moderate organ damage, but severe endothelial dysfunction, ARDS, hypercoagulation state and microangiopathy-like cytopenias [2, 26, 27]. Renal pathological findings in COVID-19 HIS are also quite unique and are characterized by tubular damage with abnormal sodium reabsorption, microangiopathy with hypoperfusion and glomerulopathy [28].

We observed that ruxolitinib improved survival in patients with a persistent fever (> 38.5 °C), which is one of the key features of COVID-19 HIS [2]. Besides fever, other clinical features of HIS, like acute renal injury or high CRP were not associated with a statistically significant superiority of ruxolitinib (probably, since they were observed in minor subsets of patients). So with the current study size, we were unable to formulate the exact HIS features where patients can benefit from ruxolitinib. However, the ability of ruxolitinib to control inflammation with endothelial damage is not unexpected. It was recently approved for steroid-refractory GVHD after allogeneic stem cell transplantation [12]. Steroid-refractory GVHD is another hyperinflammatory condition with severe endothelial dysfunction [29, 30]. The unique role of ruxolitinib in the situation of abnormal inflammation and endothelial dysfunction is confirmed by the fact that it is the first therapy approved for steroid-refractory acute GVHD in 30 years despite multiple academic studies of various immunosuppressive agents [31, 32]. Thus, it is unclear if other anti-cytokine therapies will provide similar results to JAK inhibitors.

On the contrary, case-fatality rates were comparable between dexamethasone and ruxolitinib across all subgroups of pulmonary severity defined by SpO_2_ level and computer tomography stage. Absence of difference can be explained by complex pathogenesis of lung injury in COVID-19. It was reported that besides HIS-associated interstitial edema and endothelial dysfunction, the following events may contribute to the damage: thrombosis of large and small vessels, formation of hyaline membranes, complement-associated injury, impaired surfactant production and direct viral cell injury [33–35]. Thus both dexamethasone and ruxolitinib may ameliorate HIS-associated components of lung injury, but not the others.

The major concern about anti-cytokine therapies, which was also stated in the WHO treatment guidelines [20], is the risk of secondary infections and other complications. Although the rate of hospital acquired infections varies across countries, in the studied cohort their incidence was comparable to an intensive care of other conditions [36]. Hematological toxicity is a known complication of ruxolitinib, however it is generally observed in patients with underlying hematological disease [11, 12]. In the doses used in this study, no clinically relevant hematological toxicity was observed. Although grade 1 thrombocytopenia was more prevalent after ruxolitinib, in some patients we observed, on the contrary, resolution of HIS-associated cytopenias during treatment. The reduced incidence of cardiovascular adverse events is not surprising. On the one hand, COVID-19 infection itself predisposes to venous thromboembolism, and some studies report the incidence of this complication as high as 37% [37]. On the other hand, the prothrombotic effects of steroids are known for a long time [38]. High incidence of myocardial infarction and venous thromboembolism emerged despite direct anticoagulation therapy in all patients. One of the possible explanations is the microangiopathic origin of thrombosis during COVID-19 infection, where anti-complement therapy might be a more effective way to prevent thrombotic events [39].

## Conclusion

In the general cohort of patients with severe COVID-19 infection there was not statistically significant difference in case-fatality rate between ruxolitinib as initial therapy and dexamethasone. Our results confirm a favorable safety profile seen with another JAK inhibitor, baricitinib, in the randomized trial [10]. Presumable survival benefits of ruxolitinib group were observed in the patients with a high fever (≥ 38.5 °C) and without additional cardiovascular co-morbidity. However, these findings should be proved in a separate study. The recently announced results of randomized RUXCOVID trial demonstrated no difference in the primary composite endpoint [16], but still it is unclear if this result is generalizable for all the subpopulations defined with HIS symptoms and COVID-19 severity. Hence, the final consideration about JAK inhibitors efficiency should be drawn on the basis of additional studies.

## Limitations of the study

The proposed research has the following limitations. This study was based on a patient population with COVID-19, who were undergoing in-hospital treatment in Saint-Petersburg in spring–autumn of 2020. Thus the described results refer to the disease caused by the Wuhan strain of SARS-CoV-2. Although the incidence of death was not significantly different in two groups, faster hospital discharge in the dexamethasone group may indicate that ruxolitinib was prescribed to “more severe patients” (from physicians’ subjective point of view). Thus some residual disbalance after matching could occur and dilute the effect of the therapy. This effect could be strengthened by the fact that some additional clinic features of patients could influence a doctor’s decisions regarding anti-cytokine therapy prescription but were not described in initial. Also, a few important complications of COVID-19 remained out of scope in this study and were not compared between groups. Some benefits of ruxolitinib found in high-fever and non-cardiovascular subgroups are based on unadjusted comparisons and hence should be proved in separate studies with a justified sample size.

## Supplementary Information

**Additional file 1.** Additional Figures S1–S8 and Tables S1–S7.

## Data Availability

The data that support the findings of this study are available from the corresponding author upon reasonable request.

## Abbreviations

ARDS: Acute respiratory distress syndrome
BMI: Body mass index
CI: Confidence interval
CT: Computer tomography
DIS: Disseminated intravascular coagulation
GM-CSF: Granulocyte-monocyte colony stimulating factor
GVHD: Graft-versus host disease
HIS: Hyperinflammatory syndrome
IL-1: Interleukin-1
IL-6: Interleukin-6
JAK: Januse-kinase
MAP: Managed Access Program
PCR: Polymerase chain reaction
WBC: White blood cells
WHO: World Health Organization
