# Supplementary material for: Ruxolitinib versus dexamethasone in hospitalized adults with COVID-19: multicenter matched cohort study
Source: BMC Infect Dis. 2021 Dec 22;21:1277. doi: 10.1186/s12879-021-06982-z (PMC8693127; doi:10.1186/s12879-021-06982-z)
Supplement: Supplementary file 1 — Additional file 1. Additional Figures S1–S8 and Tables S1–S7. [file 12879_2021_6982_MOESM1_ESM.docx]

Figure S1. Dose distribution in mg/kg of patient weight.


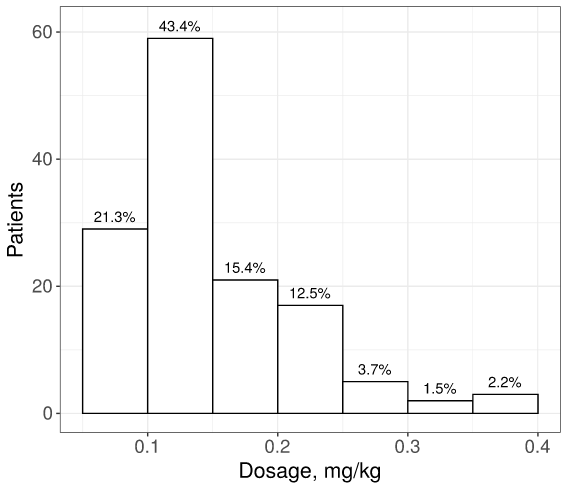


Figure S2. Covariate balance plot.


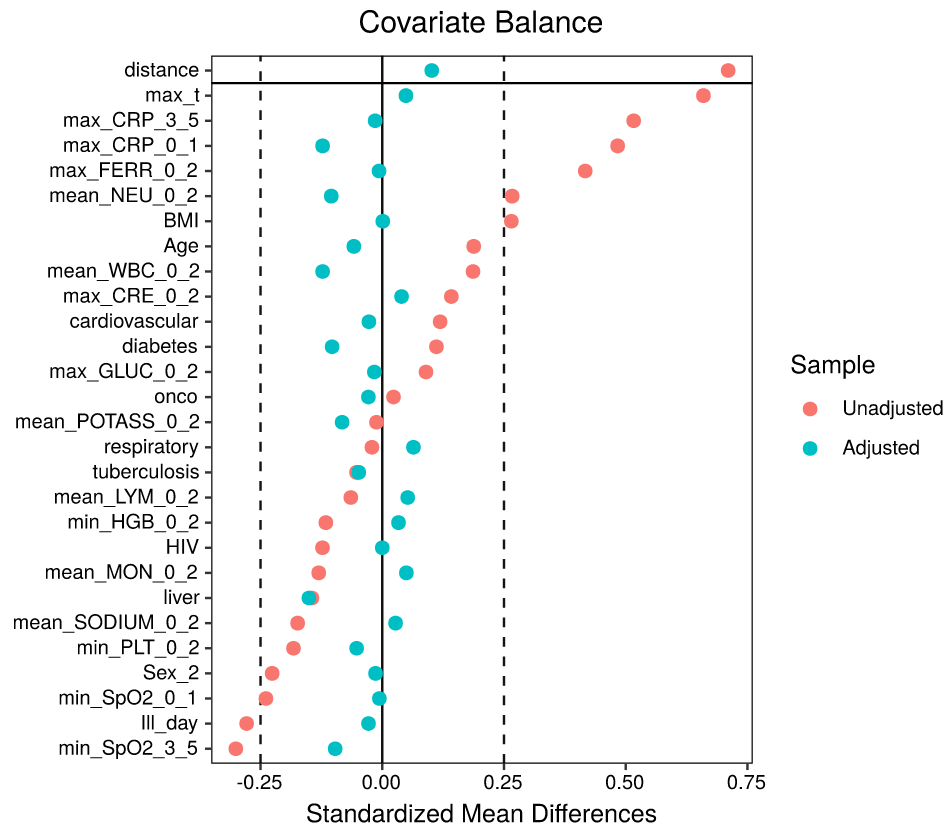


Red dots represent the standardized mean differences between the whole dexamethasone cohort (N=1355) and ruxolitinib group (N=146), while blue dots represent the difference between ruxolitinib group and 146 selected matched control patients. The vertical dotted lines indicate represent target matching quality of ±0.25. Age= age, max_t= maximal body temperature, max_CRP_0_1=maximal C-reactive protein level on 0-1 day of hospitalization, max_CRP_3_5= maximal C-reactive protein level on 3-5 day of hospitalization, mean_NEU_0_2= mean neutrophil level on 0-2 day of hospitalization, mean_WBC_0_2= mean white blood cell count on 0-2 day of hospitalization, BMI=body mass index, max_CRE_0_2= maximal creatinine level on 0-2 day of hospitalization, max_FERR_0_2= maximal ferritin on 0-2 day of hospitalization, mean_NEU_0_2= mean of neutrophils on 0-2 day of hospitalization, cardiovascular= any cardiovascular co-morbidity, max_GLUC_0_2= maximal serum glucose level on 0-2 day of hospitalization, mean_POTASS_0_2= mean serum potassium level on 0-2 day of hospitalization, mean_WBC_0_2= mean white blood cell count on 0-2 day of hospitalization, mean_MON_0_2= mean monocytes on 0-2 day of hospitalization, mean_LYM_0_2= mean lymphocytes on 0-2 day of hospitalization, onco=any oncological disease currently requiring treatment, respiratory= any concurrent chronic respiratory disease, tuberculosis= tuberculosis in the patient’s history, HIV= human immunodeficiency virus, min_HGB_0_2= minimal hemoglobin level on 0-2 day of hospitalization, min_PLT_0_2= minimal platelet level on 0-2 day of hospitalization, mean_SODIUM_0_2= mean sodium level on 0-2 day of hospitalization, liver= any concurrent chronic liver disease, Sex_2=gender, Ill_day= day of illness at the time of the study intervention, min_SPO2_0_1= minimal SpO2 without oxygen support upon admission, min_SPO2_3_5= minimal SpO2 on 3-5 day of hospitalization before study drug administration without oxygen support.

Table S1. Contingency table for group outcomes.

| Experiment | Group | Discharge | Death | OR, 95% CI | *p*-value |
| --- | --- | --- | --- | --- | --- |
| Before matching | Treatment arm | 132 | 14 | 1.95, [1.07, 3.53] | 0.036 |
|  | Reference arm | 1285 | 70 |  |  |
| After  matching | Treatment arm | 132 | 14 | 0.71, [0.31, 1.57] | 0.46 |
|  | Reference arm | 127 | 19 |  |  |

Figure S3. Sensitivity and specificity of mortality prediction according to the admission CRP levels.


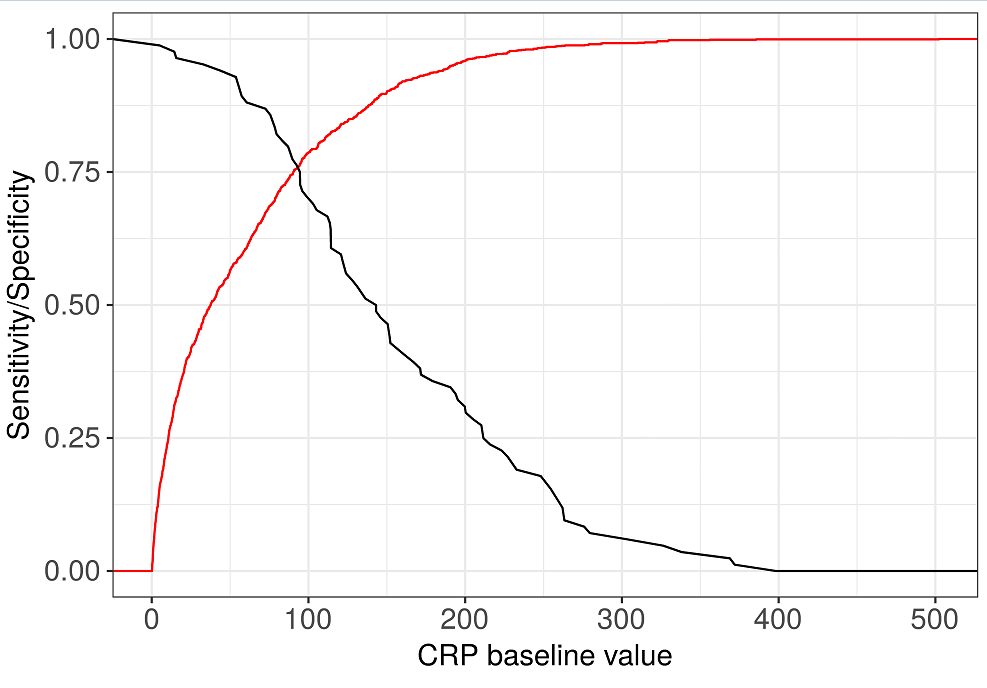


The plot is built on the subset of dexamethasone-treated patients not included in the main analysis (N=1209).

Figure S4. Cluster analysis of parameters included in the matching procedure in the ruxolitinib group.


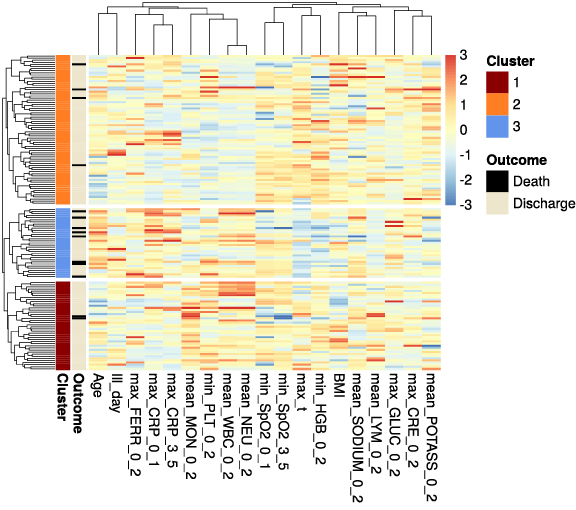


Age= age, max_t= maximal body temperature, max_CRP_0_1=maximal C-reactive protein level on 0-1 day of hospitalization, max_CRP_3_5= maximal C-reactive protein on 3-5 day of hospitalization, mean_NEU_0_2= mean neutrophil on 0-2 day of hospitalization, mean_WBC_0_2= mean white blood cell count on 0-2 day of hospitalization, mean_MON_0_2= mean monocytes on 0-2 day of hospitalization, BMI=body mass index, max_ CRE_0_2= maximal creatinine level on 0-2 day of hospitalization, max_FERR_0_2= maximal ferritin on 0-2 day of hospitalization, max_GLUC_0_2= maximal serum glucose level on 0-2 day of hospitalization, mean_POTASS_0_2= mean serum potassium level on 0-2 day of hospitalization, mean_LYM_0_2= mean lymphocytes on 0-2 day of hospitalization, min_HGB_0_2= minimal hemoglobin level on 0-2 day of hospitalization, min_PLT_0_2= minimal platelet level on 0-2 day of hospitalization, mean_SODIUM_0_2= mean sodium level on 0-2 day of hospitalization, Ill_day= day of illness at the time of the study intervention, min_SPO2_0_1= minimal SpO2 without oxygen support upon admission, min_SPO2_3_5= minimal SpO2 on 3-5 day of hospitalization - before study drug administration without oxygen support.

Table S2. Matching parameters with the clusters.

| **Names** | **Cluster 1**  **(*n* = 28)** | **Cluster 2**  **(*n* = 70)** | **Cluster 3**  **(*n* = 47)** |
| --- | --- | --- | --- |
| Age, years, mean±SD | 62.2±11.5 | 53.2±12.2 | 63.4±13.2 |
| Gender | M: 19 (67.9%) F: 9 (32.1%) | M: 41 (58.6%) F: 29 (41.4%) | M: 20 (42.6%) F: 27 (57.4%) |
| Body mass index | 31.6±5.9 | 29.2±4.9 | 31.1±6.1 |
| Day of illnes | 7±3.4 | 6.6±2.7 | 9±6 |
| Maximal creatinine, mmol/L | 0.1±0 | 0.1±0 | 0.1±0.1 |
| Admission CRP, mg/L | 123.4±82.2 | 61.9±46 | 136.1±73.7 |
| Maximal CRP, mg/L | 93.9±85.3 | 73.7±56.5 | 144.8±105.5 |
| Maximal Ferritin, ng/mL | 727.7±519.4 | 451.9±230.2 | 417.9±131.5 |
| Maximal glucose, mmol/L | 7.4±1.6 | 6.9±1.9 | 8.6±4.2 |
| Maximal temperature, °C | 38±0.9 | 38.3±1 | 37.4±0.9 |
| Mean lymphocytes, 10^9^/L | 1.3±0.5 | 1.5±0.6 | 1±0.9 |
| Minimal lymphocytes, 10^9^/L | 0.9±0.4 | 1±0.5 | 0.6±0.3 |
| Mean monocytes, 10^9^/L | 0.6±0.3 | 0.4±0.2 | 0.4±0.2 |
| Mean neutrophils, 10^9^/L | 8±4.2 | 3.7±1.4 | 5.5±2.9 |
| Minimal neutrophils, 10^9^/L | 4.6±2.2 | 2.6±1.1 | 3.1±1.6 |
| Mean WBC, 10^9^/L | 10±4.3 | 5.4±1.8 | 6.9±2.8 |
| Minimal WBC, 10^9^/L | 6.7±2.7 | 4.5±1.4 | 4.6±1.8 |
| Mean platelets, 10^9^/L | 221.4±66.2 | 181.7±58.4 | 223.6±62 |
| Minimal platelets, 10^9^/L | 183.8±75.6 | 170±53.7 | 185.6±76 |
| Admission hemoglobin, g/L | 140.1±12.3 | 142.3±16.2 | 120.6±16.7 |
| Minimal hemoglobin, g/L | 123.4±19.9 | 129.6±14.9 | 103.8±18 |
| Mean potassium, mmol/L | 3.9±0.6 | 4.1±0.4 | 4±0.5 |
| Mean sodium, mmol/L | 140.6±3.5 | 138±3 | 138.3±4.5 |
| Admission SpO2, % | 92.5±4.7 | 95.8±2 | 95.9±2.2 |
| Minimal SpO2, % | 92.8±3.6 | 94.6±3.3 | 95±3 |
| Another anticytokine therapy | Yes: 9 (32.1%) No: 19 (67.9%) | Yes: 13 (18.6%) No: 57 (81.4%) | Yes: 10 (21.3%) No: 37 (78.7%) |
| Cardiovascular disease | Yes: 18 (64.3%) No: 10 (35.7%) | Yes: 25 (35.7%) No: 45 (64.3%) | Yes: 27 (57.4%) No: 20 (42.6%) |
| Diabetes | Yes: 6 (21.4%) No: 22 (78.6%) | Yes: 9 (12.9%) No: 61 (87.1%) | Yes: 13 (27.7%) No: 34 (72.3%) |
| Atrial fibrillation | Yes: 4 (14.3%) No: 24 (85.7%) | Yes: 4 (5.7%) No: 66 (94.3%) | Yes: 4 (8.5%) No: 43 (91.5%) |
| HIV | Yes: 0 (0%) No: 28 (100%) | Yes: 0 (0%) No: 70 (100%) | Yes: 1 (2.1%) No: 46 (97.9%) |
| Ongoing immunosupressive therapy for concurrent disease | Yes: 0 (0%) No: 28 (100%) | Yes: 0 (0%) No: 70 (100%) | Yes: 0 (0%) No: 47 (100%) |
| Chronic liver disease | Yes: 0 (0%) No: 28 (100%) | Yes: 2 (2.9%) No: 68 (97.1%) | Yes: 3 (6.4%) No: 44 (93.6%) |
| Oncological treatment | Yes: 1 (3.6%) No: 27 (96.4%) | Yes: 3 (4.3%) No: 67 (95.7%) | Yes: 4 (8.5%) No: 43 (91.5%) |
| Chronic respiratory disease | Yes: 4 (14.3%) No: 24 (85.7%) | Yes: 4 (5.7%) No: 66 (94.3%) | Yes: 8 (17%) No: 39 (83%) |
| Tuberculosis in the patient’s history | Yes: 0 (0%) No: 28 (100%) | Yes: 1 (1.4%) No: 69 (98.6%) | Yes: 2 (4.3%) No: 45 (95.7%) |

Table S3. Contingency tables for detected clusters

| Cluster | Group | Discharge | Death | OR, 95% CI | *p*-value | adj. *p* |
| --- | --- | --- | --- | --- | --- | --- |
| 1 | Treatment arm | 24 | 4 | 0.51, [0.095, 2.33] | 0.5 | 1 |
|  | Reference arm | 21 | 7 |  |  |  |
| 2 | Treatment arm | 69 | 1 | 0.19, [0.004, 1.77] | 0.21 | 0.63 |
|  | Reference arm | 65 | 5 |  |  |  |
| 3 | Treatment arm | 41 | 6 | 0.32, [0.09, 0.98] | 0.046 | 0.138 |
|  | Reference arm | 32 | 15 |  |  |  |

Figure S5. Covariate balance for detected clusters.


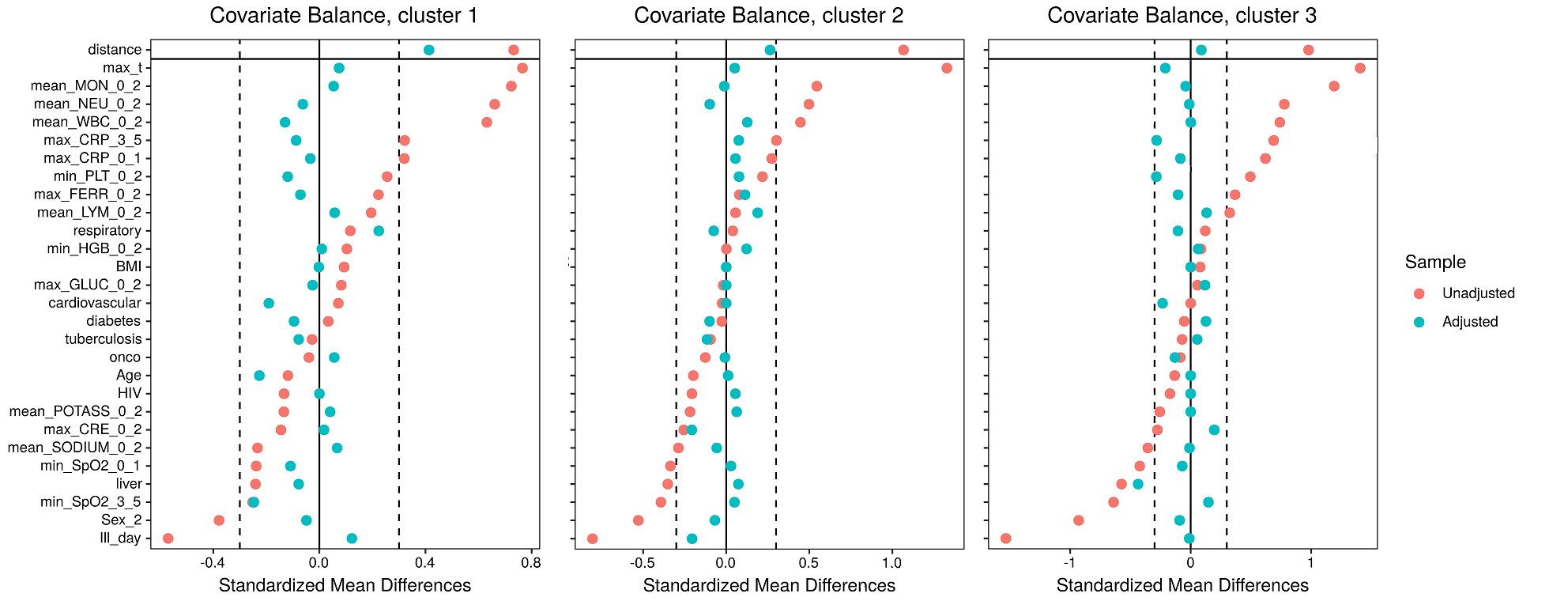


Table S4. List of main *R* packages used in the project

| Packages | Purpose | Reference |
| --- | --- | --- |
| ggplot, ggpubr | General data visualization. | [1-2] |
| dplyr, tidyr | Data preprocessing. | [3-4] |
| cr17 | Survival analysis | [5] |
| pheatmap | Heatmaps visualization and clustering. | [6] |
| NbClust | Choice of appropriate number of clusters. | [7] |
| MatchIt, cobalt | Propensity score matching and covariate balance visualization. | [8] |
| farringtonManning | Farrington-Manning test for non-inferiority analysis | [9] |

1. H. Wickham. ggplot2: Elegant Graphics for Data Analysis. Springer-Verlag, New York, 2016.

2. Alboukadel Kassambara (2019). ggpubr: 'ggplot2' Based Publication Ready Plots. R package version 0.2.4. https://CRAN.R-project.org/package=ggpubr

3. Hadley Wickham, Romain François, Lionel Henry and Kirill Müller (2020). dplyr: A Grammar of Data Manipulation. R package version 1.0.2. https://CRAN.R-project.org/package=dplyr

4. Hadley Wickham and Lionel Henry (2019). tidyr: Tidy Messy Data. R package version 1.0.0. https://CRAN.R-project.org/package=tidyr

5. cr17: Testing Differences Between Competing Risks Models and Their Visualisations. https://CRAN.R-project.org/package=cr17

6. Raivo Kolde (2019). pheatmap: Pretty Heatmaps. R package version 1.0.12. https://CRAN.R-project.org/package=pheatmap

7. Malika Charrad, Nadia Ghazzali, Veronique Boiteau, Azam Niknafs (2014). NbClust: An R Package for Determining the Relevant Number of Clusters in a Data Set. Journal of Statistical Software, 61(6), 1-36. http://www.jstatsoft.org/v61/i06/

8. Daniel E. Ho, Kosuke Imai, Gary King, Elizabeth A. Stuart (2011). MatchIt: Nonparametric Preprocessing for Parametric Causal Inference. Journal of Statistical Software, Vol. 42, No. 8, pp. 1-28. URL https://www.jstatsoft.org/v42/i08/

9. Kevin Kunzmann (2015). farringtonManning: Testing for rate differences. R package version 0.2. https://github.com/kkmann/farringtonManning/

Figure S6. Subgroup analysis according to the physical characteristics


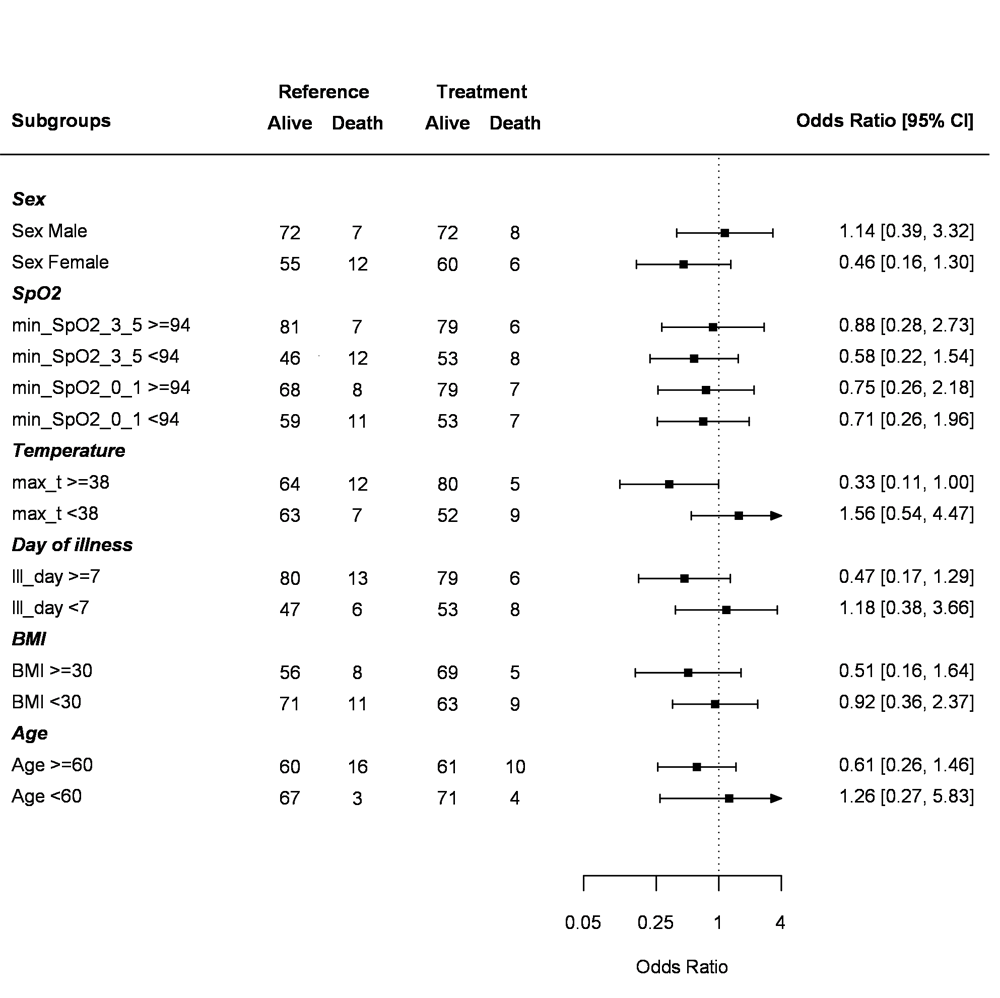


Figure S7. Subgroup analysis according to the admission biochemistry parameters


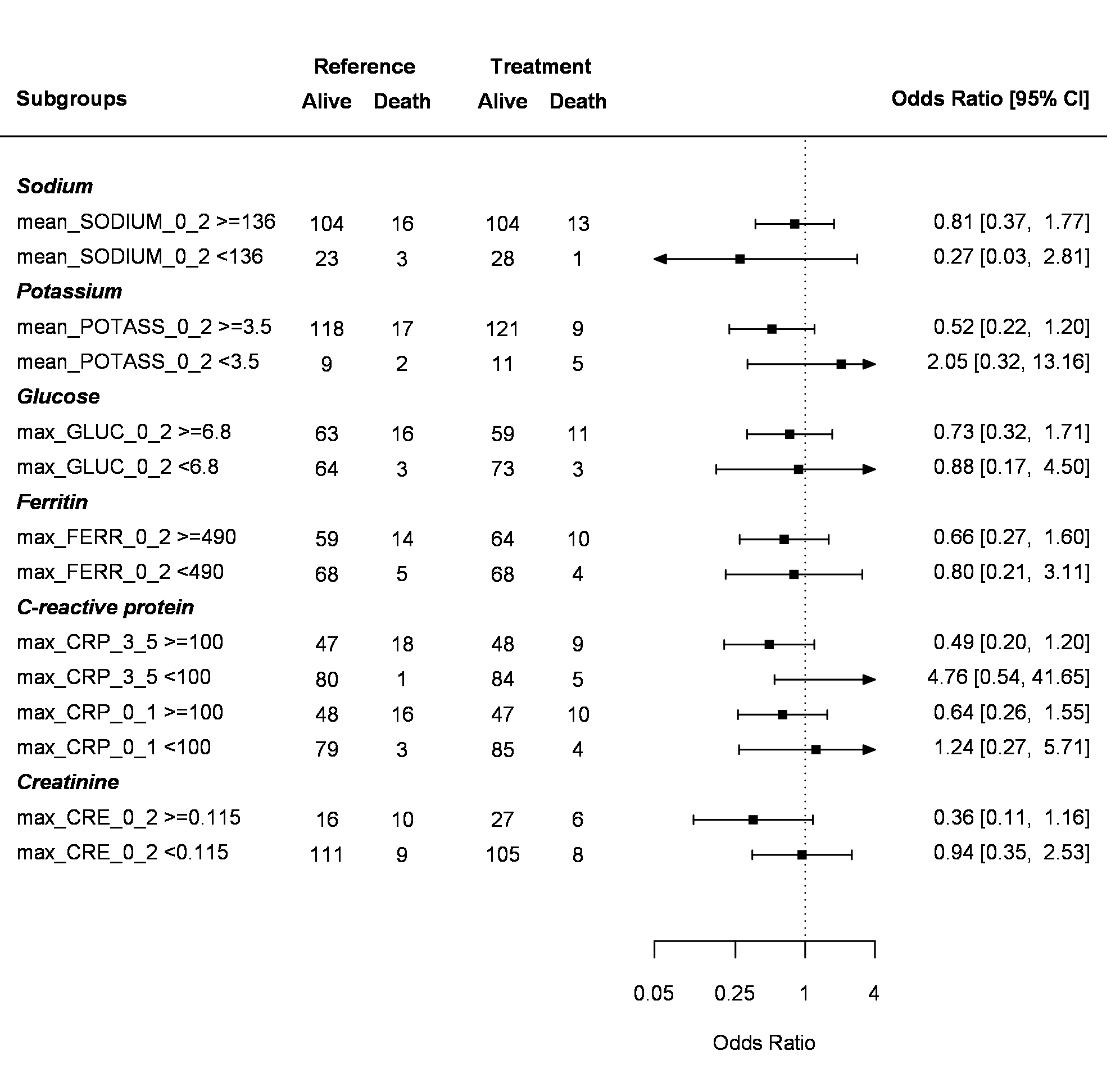


Figure S8. Subgroup analysis according to the co-morbidities.


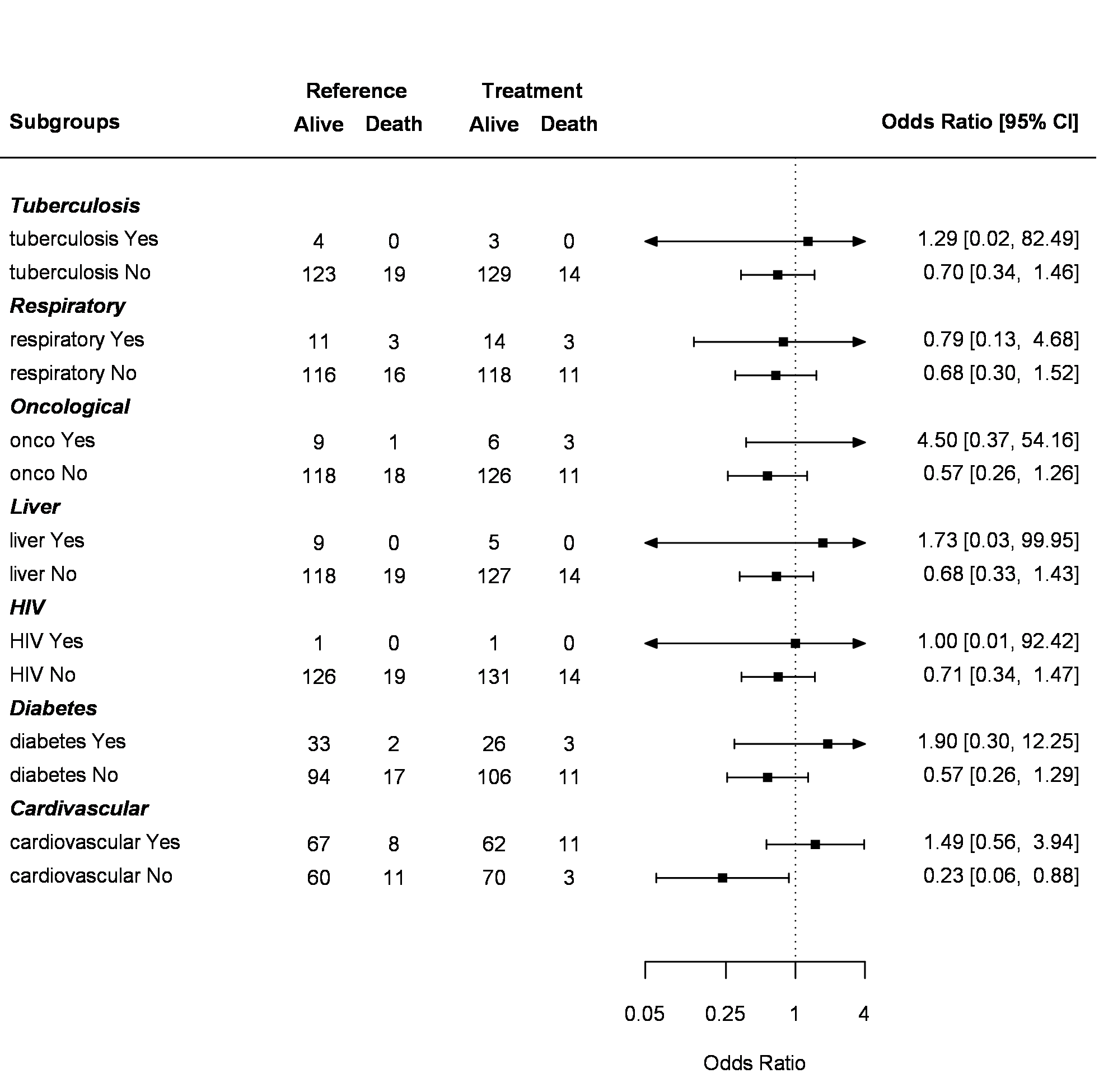


Table S5. Comparison of ruxolitinib and dexamethasone across COVID-19 severity based on computer tomography evaluation. Odds ratio below 1.0 is in favor of ruxolitinib, above 1.0 – in favor of dexamethasone.

| Computer tomography stage | Odds ratio (OR) | OR 95% CI | P-value |
| --- | --- | --- | --- |
| Stage 1 (<25%) | N/A (no cases of mortality) | N/A (no cases of mortality) | 1.0 |
| Stage 2 (25-50%) | 0.32 | 0.17-1.9 | 0.56 |
| Stage 3 (51-75%) | 0.59 | 0.37-2.13 | 0.59 |

Table S6. Incidence of hematological adverse events in the ruxolitinib (treatment) and dexamethasone (reference) arms.

| Test | Group | 0 | 1 | 2 | 3 | 4 | *p* |
| --- | --- | --- | --- | --- | --- | --- | --- |
| HGB | Reference | 56 (38.4%) | 67 (45.9%) | 15 (10.3%) | 7 (4.8%) | 1 (0.7%) | 0.69 |
|  | Treatment | 50 (34.2%) | 71 (48.6%) | 20 (13.7%) | 5 (3.4%) | 0 (0%) |  |
| LYM | Reference | 42 (28.8%) | 46 (31.5%) | 32 (21.9%) | 26 (17.8%) | 0 (0%) | 0.15 |
|  | Treatment | 27 (18.5%) | 46 (31.5%) | 42 (28.8%) | 29 (19.9%) | 2 (1.4%) |  |
| PLT | Reference | 107 (73.3%) | 34 (23.3%) | 4 (2.7%) | 1 (0.7%) | 0 (0%) | 0.042 |
|  | Treatment | 86 (58.9%) | 54 (37%) | 4 (2.7%) | 1 (0.7%) | 1 (0.7%) |  |
| WBC | Reference | 109 (74.7%) | 24 (16.4%) | 10 (6.8%) | 3 (2.1%) | 0 (0%) | 0.33 |
|  | Treatment | 94 (64.4%) | 32 (21.9%) | 14 (9.6%) | 5 (3.4%) | 1 (0.7%) |  |

Table S7. Rare adverse events and their incidence

| Ruxolitnib arm | Incidence, N(%) | Dexamethasone arm | Incidence, N(%) |
| --- | --- | --- | --- |
| Oral candidiasis | 2 (1.4%) | Acute kidney injury | 2 (1.4%) |
| Herpes Zoster | 1 (0.7%) | Purulent cholecystitis | 1 (0.7%) |
| Toxic hepatitis grade 3 | 1 (0.7%) | Purulent otitis | 1 (0.7%) |
| Gastrointestinal bleeding | 1 (0.7%) | Acute pancreatitis | 1 (0.7%) |
| Spontaneous intra-abdominal hemorrhage | 1 (0.7%) |  |  |
